# Overcome the Fear of Exercise in Patients With Bone Metastases: A Qualitative Study on Patients' Perception

**DOI:** 10.1002/cam4.70865

**Published:** 2025-04-17

**Authors:** Anita Borsati, Daniela Tregnago, Matteo Caleffi, Christian Ciurnelli, Linda Toniolo, Ilaria Trestini, Lorenzo Belluomini, Marco Sposito, Jessica Insolda, Federico Schena, Michele Milella, Sara Pilotto, Alice Avancini

**Affiliations:** ^1^ Biomedical, Clinical and Experimental Sciences, Department of Medicine University of Verona Verona Italy; ^2^ Department of Neurosciences, Biomedicine and Movement University of Verona Verona Italy; ^3^ Section of Innovation Biomedicine – Oncology Area, Department of Engineering for Innovation Medicine (DIMI) University of Verona and University and Hospital Trust (AOUI) of Verona Verona Italy; ^4^ Dietetic Service, Medical Direction Azienda Ospedaliera Universitaria Integrata di Verona Verona Italy

**Keywords:** barriers, bone metastases, exercise, facilitators, needs, perception

## Abstract

**Purpose:**

This qualitative study aimed to explore the experiences of patients with cancer and bone metastases who participated in a personalized exercise program.

**Methods:**

Individual interviews were conducted through purposeful sampling of patients who attended the 3‐month supervised exercise program. Using a phenomenological approach, semi‐structured questions were developed to investigate the benefits, risks, facilitators, and barriers related to exercise. Data were analyzed using inductive thematic analysis.

**Results:**

Thirteen patients with mixed cancer types participated in the study. Patients recognized the physical and psychological benefits of exercise, such as improvements in physical fitness, bone health, reduction of symptoms, especially pain and fatigue, and increase in self‐confidence. While they did not perceive any disadvantage from participating in the program, they acknowledged the risk of bone fractures or exacerbation of lesions if the intervention is not tailored and supervised. The program's structure, including the involvement of dedicated specialists, has been reported as a fundamental pillar. Among the modulators for participation and adherence, comorbidities associated with bone metastases, logistic barriers, and lack of social support may be obstacles. Conversely, recommendations from the oncologist, personal motivation, and peer support were found to be facilitators of practice.

**Conclusions:**

Patients with bone metastases expressed a broad range of benefits from participation in a structured exercise program. Several modulators may affect uptake and adherence and thus should be considered when designing a dedicated program.

## 1 Introduction

Due to the molecular properties of bone tissue, cancer cells from solid tumors frequently tend to metastasize to the bones. Bone metastases represent a debilitating stage of the disease associated with an increase in morbidity and mortality [1], and advanced breast (70%), prostate (85%), lung (40%), and kidney (40%) carcinomas are the most affected malignancies [2]. Patients with bone metastases may face a high risk of skeletal‐related complications such as pain, pathological fractures, or spinal cord injuries, as well as reduced physical function, which can severely impact their quality of life [3].

In this complex and impactful setting, physical exercise has been proposed as a safety strategy to maintain a patient's physical condition, manage symptoms, such as fatigue and pain, enhance the quality of life, and improve bone health [4, 5, 6]. In this sense, a randomized controlled trial examining the effects of 12 weeks of isometric resistance training on 60 patients with spinal bone metastases found significant improvements in bone density at the bone lesion sites. Particularly at 3 and 6 months, the interventional group exhibited an increase in the bone metastases density of 28.3% and 80%, respectively, while the controls did not report any significant changes [7]. However, these promising findings require validation in larger studies. In this sense, two trials are currently evaluating exercise programs on skeletal health in women with breast cancer and stable osteolytic metastases and in men with advanced prostate cancer and stable sclerotic spinal metastases [8, 9].

In 2022, the International Bone Metastasis Exercise Working Group published the best practice recommendations to help exercise specialists and healthcare providers integrate physical exercise safely into caring for patients with bone lesions [8, 10]. These recommendations enclose a series of indications, such as the need to identify patients at high risk of exercise‐related skeletal complications, consult with a medical team, and involve an exercise specialist experienced in managing bone metastases to prescribe and adapt appropriate assessments and training [10]. In addition, regarding the exercise prescription, they suggest following the current exercise guidelines for patients with cancer, that is, three times per week of aerobic exercise, lasting 30 min per session at moderate intensity, plus resistance training twice a week, performed in two to three sets of 8–12 repetitions [8]. However, despite the benefits and the published guidelines, most patients with bone metastases are insufficiently active [9, 11, 12]. Although several barriers that might interfere with physical exercise participation in the bone metastatic setting have been identified, one of the most common concerns that may discourage patients from engaging in regular physical activity remains the fear of injury [13, 14]. Indeed, although the safety of physical exercise, especially in a supervised context, has been demonstrated [4, 5, 15], studies highlight that fear of skeletal‐related events, pain, and the lack of dedicated professionals remain reasons for the low participation [13, 16]. On the other hand, 53% of healthcare providers felt unconfident in recommending physical exercise in the bone metastatic setting, especially due to safety issues [17]. Nevertheless, most available evidence regarding perceptions, barriers, and facilitators in the bone metastatic context enclosed patients who did not participate in physical exercise programs. Understanding the experiences of patients who engage in an individualized exercise intervention may be equally important in optimizing and further tailoring the prescription. To address this gap, the current study aimed to explore the experiences and perceptions of patients affected by bone metastases who have performed a tailored and supervised exercise intervention.

## 2 Materials and Methods

### 2.1 Study Design

A qualitative study design, using a phenomenological approach, was applied to explore the perceptions of patients with bone metastases regarding their engagement in physical exercise. The inductive interpretative approach was employed to capture the lived experiences of patients and develop a conceptual model to improve exercise program implementation for this population. The study protocol adhered to Good Clinical Practice principles, and all procedures were conducted in compliance with the Helsinki and Oviedo declarations. The Ethics Committee for Clinical Trials (Prot. N. 33320) approved the study. The current investigation was reported according to the Consolidated Criteria for Reporting Qualitative Research (COREQ) guidelines [18].

### 2.2 Context and Sampling

The study took place between September 2023 and June 2024 at the Department of Neuroscience, Biomedicine, and Movement of the University of Verona in Italy. A purposive sampling method was used to recruit patients affected by bone metastatic disease who had performed a personalized and supervised exercise program and who varied in age, gender, and cancer site. Eligibility criteria included being over 18 years old, having one or more bone metastases, participating in the exercise program, and consenting to be interviewed. Participants were selected as “rich cases” due to their direct involvement in the exercise program, which provided valuable and unique insights. Sampling continued until data saturation was achieved, with no new information emerging during interviews.

### 2.3 The Exercise Program

Patients interviewed have participated in a 12‐week tailored exercise program at the University of Verona, supervised by expert kinesiologists with working experience in patients with bone metastases [19]. The intervention included bi‐weekly sessions of combined aerobic and resistance training. Briefly, the aerobic component started with 10–15 min and gradually increased to 25–30 min at moderate intensity, set with the 10‐point Borg Rating of Perceived Exertion Scale. The resistance part consisted of six total body exercises using free weights or elastic bands performed at moderate intensity. Each activity was specifically adapted to the type and location of their bone metastases by expert kinesiologists, and in accordance with the current recommendations [10], patients were instructed on proper exercise techniques, and pain at the level of bone lesions was strictly monitored during each session. Additional information about the program is available in the Supporting Information.

### 2.4 Data Collection and Study Procedures

Individual interviews were held using the online platform Zoom [20]. Based on previous studies [13, 21], semi‐structured interview questions were developed to investigate patients' perspectives about benefits, risks, facilitators, and barriers related to exercise according to the Health Belief Model (Table 1) [22]. The Health Belief Model assumes that people are likely to adopt a behavior if they perceive themselves to be at risk of developing a condition (perceived susceptibility), believe the condition could have serious consequences (perceived severity), think that a specific course of action would reduce their risk or severity of the condition or bring about other positive outcomes (perceived benefits), and see few obstacles to the health action (perceived barriers). Additionally, the model suggests that specific cues, such as environmental factors, can influence the final action taken.

**TABLE 1:** Questions and prompts that guided the interview.

Categories	Questions
Benefits of exercise	In your opinion, what are the benefits of an exercise program in the context of cancer? What benefits do you think it has for bone structure? How about regarding the presence of bone metastases?
Disadvantages/risks of exercise	In your opinion, what do you consider to be the disadvantages or potential risks of an exercise program in an oncological context? What disadvantages/risks do you think it has for bone structure? How about regarding the presence of bone metastases?
Facilitators for exercise	In your opinion, what are the factors that might facilitate participation in an exercise program for patients with cancer? Considering personal, interpersonal (e.g., physician, family members), and environmental factors (e.g., access to facilities, location, clinical setting), are there specific factors that would encourage participation among individuals with bone fragility? Who would you prefer to receive such information from, and in what manner?
Barriers for exercise	In your opinion, what are the factors that might hinder participation in an exercise program for patients with cancer? Considering personal, interpersonal (e.g., physician, family members), and environmental factors (e.g., access to facilities, location, clinical setting), are there specific factors that would discourage participation among individuals with bone fragility?

Each interview lasted approximately 30–45 min and was recorded and transcribed verbatim. Pseudonyms were used to report the data to ensure confidentiality. Sociodemographic data (birth date, education level, marital status, occupational status, and family income), clinical variables (cancer type and stage, treatment status, and site of bone metastases), and physical activity levels were collected using a Google form completed by the participants. The current exercise level was assessed using the Godin Leisure‐Time Exercise Questionnaire, which inquired about the frequency of vigorous, moderate, and mild‐intensity exercise during the previous week's leisure time [23].

### 2.5 Research Team and Reflexivity

All interviews were conducted by DT, a psychologist from the Oncology Unit at the University of Verona Hospital Trust, ensuring consistency in moderation. DT was not involved in the exercise program to minimize potential bias in participant responses and let them be free to express their opinions. MC, a graduate student specializing in preventive and adapted physical activity, assisted during the sessions and recorded the discussions. The multidisciplinary research team included professionals deeply involved in participant care: four kinesiologists with expertise in exercise oncology (AB, LT, CC, and AA), one registered dietician working in the oncology day hospital (IT), four medical oncologists (LB, MS, MM, and SP), one data manager (JI), and one sports physician (FS). All team members have experience in conducting qualitative research, which enriched the study design and data analysis processes. To further enhance reflexivity, the qualitative analysis was conducted by a researcher who did not directly train the participants. Additionally, any discrepancies in data interpretation were resolved through discussion with a third external team member.

### 2.6 Analysis

AB and MC independently conducted a thematic analysis of the transcripts following Braun and Clarke's methodology, using the Atlas.ti software [24]. Initially, they read through the entire text multiple times to gain an overall understanding and familiarize themselves with the content. Next, they identified meaning units and developed corresponding codes. In the third phase, these codes were organized and grouped into themes and sub‐themes. Afterward, the themes and sub‐themes were reviewed and refined concerning the coded extracts and the complete data set. Finally, AB and AA discussed the findings, compared sub‐themes and themes, resolved any disagreements, and finalized and named the themes. Descriptive statistics were employed to analyze the questionnaire data, encompassing demographic and clinical characteristics and physical activity levels. Patients were categorized as either “active” or “insufficiently active” based on the Leisure Score Index (LSI). This index is calculated by summing the product of weekly exercise frequencies for vigorous activities (multiplied by 9) and moderate activities (multiplied by 5) [25, 26].

### 2.7 Techniques to Enhance Trustworthiness

To ensure the trustworthiness of the qualitative research, the four criteria of Lincoln and Guba were followed: credibility, transferability, confirmability, and dependability [27]. Credibility was strengthened through prolonged engagement with study participants and independent analysis by three clinical researchers (AB, MC, and AA) with experience in oncology. The interviews were conducted within three months of completing the exercise program to minimize recall bias and ensure an accurate collection of participants' experiences. Moreover, reflexivity was employed to minimize personal bias, guaranteeing an objective interpretation of data. Confirmability was enhanced by peer debriefing and collaboration with a multidisciplinary team, including kinesiologists, both involved and not involved in the exercise program, psychologists, and oncologists. This diversity mitigated researcher bias and enriched the findings. Dependability was ensured by maintaining a detailed audit trail, with data rigorously reviewed during collection and analysis. A clear coding schema was developed, identifying patterns and codes, and a third reviewer validated the findings to promote replicability. Lastly, transferability was supported by providing a detailed description of the study context, procedures, and findings, enabling applicability in similar settings. This comprehensive documentation allows future researchers to assess the study's relevance to their contexts.

## 3 Results

### 3.1 Participants

Initially, 19 patients were approached and invited to participate in the study, and of those, 13 accepted to be interviewed. Lack of interest and unavailability on scheduled days were the main reasons for refusing. Unfortunately, one patient died after completing the interview and did not complete the questionnaires. For this reason, Table 2 displays the socio‐demographic and clinical characteristics of 12 patients. The large majority of the respondents were female (75%), had a mean age of 59.3 (±10.3), and 83% had a high school degree. Breast (*n* = 4), lung (*n* = 4), and prostate (*n* = 3) cancer were the most represented tumors, and 92% were currently receiving anti‐cancer treatments, mainly radiotherapy and chemotherapy. Regarding bone metastases, 83% of participants presented lesions at the spine, 33% at the pelvis, and 33% at the proximal femur.

**TABLE 2:** Socio‐demographic and clinical characteristics of study participants.

Characteristics (*n* = 12)	*N* (%)
Sex	
Male	3 (25)
Female	9 (75)
Educational level	
Secondary	2 (17)
High school degree	10 (83)
Marital status	
Married	12 (100)
Employment	
Full‐time employed	2 (17)
Part‐time employed	2 (17)
Retired	8 (67)
Family income	
More than adequate	7 (58)
Adequate	2 (17)
Barely adequate	3 (25)
Cancer site	
Breast	4 (33)
Lung	4 (33)
Prostate	3 (25)
Colorectal	1 (8)
Bone metastases sites	
Spine	10 (83)
Ribs/sternum	3 (25)
Pelvis	4 (33)
Proximal femur/humerus	4 (33)
Type of treatment	
Surgery	4 (33)
Chemotherapy	6 (50)
Radiotherapy	7 (58)
Hormone therapy	5 (42)
Immunotherapy	2 (17)
Current treatment status	
Ongoing	11 (92)
Ended	1 (8)
Physical activity level	
Active (LSI ≥ 24)	7 (58)
Insufficient active (LSI < 24)	5 (42)

### 3.2 Thematic Findings

The thematic analysis identified six main themes, each comprising a series of subthemes that emerged from the interviews, culminating in the development of a conceptual model to explain the perceived impact of a tailored exercise program on patients with bone metastases and the factors influencing their participation and adherence. Overall, the themes were structured as follows (i) bone metastatic disease affects patients' lives, (ii) exercise may reinforce the body and mind, (iii) and impact bone health, (iv) a structured and tailored program is the key, (v) personal/interpersonal, and (vi) environmental/external modulators may influence the engagement.

**FIGURE 1:**
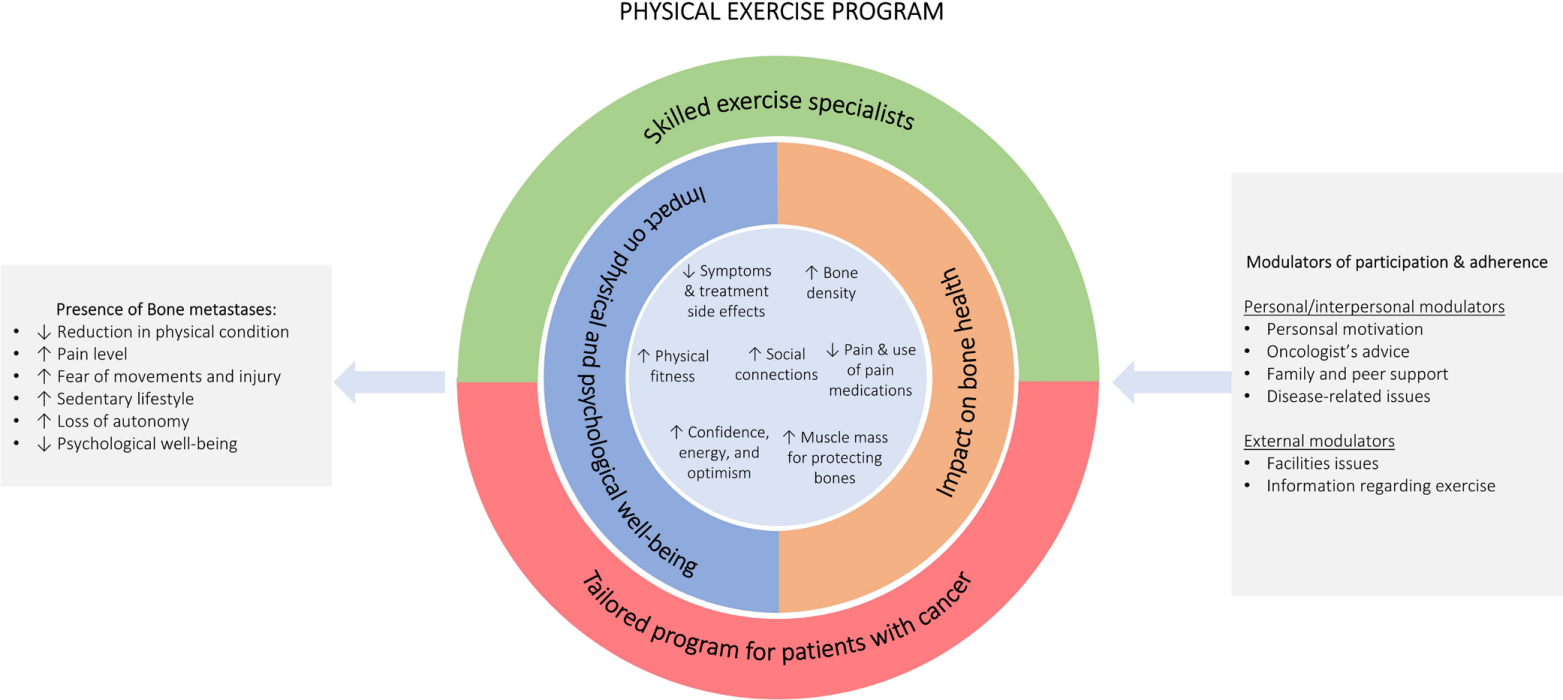
Benefits of exercise, exercise program, and modulators for participation and adherence.

The model (Figure 1) starts with the background that bone metastatic disease and related side effects profoundly impact the psycho‐physical well‐being of patients, disrupting daily routines and weakening their confidence in movement. Exercise may be a supportive care strategy for ameliorating the impairment caused by cancer, treatments, and bone metastases. In this sense, patients participating in a supervised exercise program stated that it was a valuable experience for regaining physical fitness, ameliorating symptoms, improving bone health despite some misconceptions regarding safety issues, and enhancing psychological well‐being. The identified key to achieving benefits and minimizing the potential injury risks was the structure of the program, specifically designed for patients with cancer, with dedicated exercise professionals able to tailor the prescription according to physical and disease conditions and adapt it to bone metastases. Nevertheless, compliance and adherence to the program are fundamental for expecting benefits. In this regard, participants identified a series of personal features, including personal attitude, oncologist recommendation, family and peer support, treatment side effects, and external issues, such as access to facilities and information regarding exercise, that may positively or negatively modulate the willingness and engagement of patients in exercise.

### 3.3 Theme 1: Bone Metastatic Disease Affects Patients' Lives

Patients reported that the combined impact of bone metastases and treatment side effects disrupted their physical abilities, heightened their fear of movement, and altered their sense of normalcy. Patients revealed that bone metastases impose significant physical burdens, often resulting in severe limitations in daily activities. They described the persistent pain caused by metastases as debilitating, rendering even basic movements excruciating. This pain, often exacerbated by treatments such as hormone therapy and associated with the fear of injury related to movements, led to physical deconditioning, muscle loss, and an increased tendency toward a sedentary lifestyle.

I usually attended my hospital appointments with a wheelchair. I only got up from the couch to go to the bathroom. I couldn't walk. I was walking very badly and in excruciating pain. (Valentina, lung cancer, metastases at the spine, pelvis, and ribs)

The loss of mobility resulted in a sharp decline in independence, leaving them unable to perform household tasks or other routine responsibilities. In turn, this loss of autonomy deeply affected their emotional well‐being. Patients emphasized how their lives had changed irreversibly; many expressed feelings of vulnerability, sadness, frustration, and grief over the realization that they were no longer the same as they had been before the diagnosis, and patients who were physically active before found these changes particularly challenging.

Bone metastases limited me in so many things…, if before I walked 20 kilometers, now I do 4 kilometers, or if I wear my corset I do only 2 kilometers…this has a psychological impact. (Paola, lung cancer, metastases at the spine and femur)

### 3.4 Theme 2: A Physical Exercise Program May Reinforce the Body and Mind

Patients consistently recognized the psycho‐physical benefits gained from participating in the exercise program. Two distinct subthemes emerged from the interviews: (i) improvement in physical fitness, symptoms, and activities of daily living and (ii) not letting bone metastases stop you—psycho‐social well‐being.

#### 3.4.1 Subtheme 2.1: Improvement in Physical Fitness, Symptoms, and Activities of Daily Living

Patients reported that cancer, its treatments, and the presence of bone metastases lead to a reduction in overall physical activity and consequently to physical deterioration, exacerbating symptoms. Nevertheless, participants described how the exercise program interrupted this cycle, allowing them to regain their physical fitness. They stated that exercise served as a means of addressing muscle weakness, as well as symptoms such as breathing difficulties, muscular problems, and cancer‐related fatigue. Several participants perceived the paradoxical nature of exercise, that is, although it is an activity that requires effort and can initially feel exhausting, it ultimately helps alleviate chronic fatigue when done consistently.

When I walk on the treadmill, I initially feel a sense of fatigue, but then you feel that your heart starts to beat more regularly, and as I continue walking, I feel that the fatigue decreases. (Francesca, lung cancer, metastases at the spine)

Another significant benefit reported by participants was the ability to resume daily activities that had previously been challenging or impossible due to bone metastases. The regained ability to perform routine activities such as tying shoes, getting in and out of a car, and walking was seen as transformative and impactful on overall well‐being and, thus, on their quality of life. Patients emphasized that exercise taught them proper techniques to perform movements safely and effectively, even with bone frailty.

It is clear that I do not have to lift heavy weights, but in the daily routine, I could face lifting an armchair or a shopping bag. Even knowing I have frail bones, I can do it because I am trained to do it correctly. For me, it is essential to bring a certain type of movement back into normal life. (Alberto, prostate cancer, metastases at the spine and humerus)

#### 3.4.2 Subtheme 2.2: Not Letting Bone Metastases Stop You: The Psycho‐Social Well‐Being

Engaging in exercise distracted the patients' minds, helping to shift their focus away from the constant awareness of their illness and associated worries. This mental break was described as crucial for alleviating moments of anguish and sadness often triggered by their condition, offering a sense of relaxation.

Exercise is also good for the mind because, in my medical situation, I need it… the moment of anguish is always around the corner. (Riccardo, prostate cancer, metastases at the spine, ribs, and pelvis)

Patients described exercise as a coping strategy that instills positivity and a source of renewed energy and optimism, enabling them to approach the rest of their day with a more positive mindset. By engaging in exercise, patients felt they were actively contributing to their health, which was a significant source of motivation. Participation in the exercise program helped patients regain a sense of control over their lives, helping to rebuild confidence in their movements previously damaged by the presence of bone metastases. They reported a psychological shift, perceiving themselves as capable and resilient individuals rather than passive sufferers of their condition. Some participants shared how exercise allowed them to feel less like “*patients with cancer*,” breaking away from the stereotype of frailty and inactivity.

I feel reborn… when I went out, I usually had to keep my head down to avoid stumbling, but now I feel your body is strength. I am capable of walking without constantly worrying that I might break something. (Angelica, lung cancer, metastases at the spine, pelvis, and femur)

Beyond these advances, exercise also provided opportunities for social connection, which became a valuable aspect of the program. Participants appreciated the chance to meet other patients facing similar challenges, fostering a sense of community and shared experience. Some patients highlighted the meaningful friendships formed through the program's participation, particularly with those who understood the unique struggles of living with bone metastases.

### 3.5 Theme 3: Exercising May Impact Bone Health

Patients reflected on the dual nature of exercise related to bone metastases and health, recognizing its potential benefits and risks. Three subthemes emerged from the interviews: (i) amelioration of pain caused by bone metastases, (ii) enhancement of bone health, and (iii) risk of increasing skeletal‐related adverse events and disease progression.

#### 3.5.1 Subtheme 3.1: Amelioration of Pain Caused by Bone Metastases

From interviews, a reduction in the perceived pain levels emerged. This outlook was stated by several participants who recognized exercise as a way to counteract the pain from bone metastases and the side effects of anticancer treatments. For some, the improvements in pain were so substantial that they reported having reduced or entirely discontinued their reliance on pain medications.

By exercising, I slowly managed to eliminate all the anti‐inflammatories and opium pills I was taking. (Valentina, lung cancer, metastases at the spine, pelvis, and ribs)

#### 3.5.2 Subtheme 3.2: Enhancement of Bone Health

The understanding of how exercise influences bone health varied significantly among patients, with some expressing uncertainty about its effects. Several participants admitted they did not know whether exercise could directly benefit their bones, probably reflecting a lack of awareness about the relationship between physical activity and skeletal health. Others speculated that exercise may improve bone health through increasing muscle mass and directly act on bone density, even if a deepening understanding of the underlying impact remains less known.

In my opinion, the bones also strengthen because they are stimulated by physical exercise…it is beneficial for increasing my bone density. (Maria, breast cancer, metastases at the spine and femur)

#### 3.5.3 Subtheme 3.3: Risk of Increased Skeletal‐Related Adverse Events and Disease Progression

Patients were also aware of the risks of improper or unsupervised physical activity. Patients frequently cited safety as a key consideration, expressing concerns about the possibility of fractures or exacerbation of bone pain if exercises were not tailored to their specific condition.

If exercise is not tailored to each person, and for example comprises activities that excessively stress the lesion, it could expose to fracture risk. (Luca, colorectal cancer, metastases at the spine)

My neck pain had returned, and my thought was that the disease had worsened… then, feeling that things were returning to normal, I calmed down. So, I was probably moving in the wrong way. Movements done incorrectly lead you to move what you should not. (Francesca, lung cancer, metastases at the spine)

Participants also raised concerns about the broader implications of exercise on their disease progression. Two patients expressed the view that, in some cases, exercise could be contraindicated because it might worsen metastases or induce disease progression. Despite these concerns, nearly all patients who participated in supervised exercise programs expressed a strong sense of safety and trust in the tailored interventions they received.

For me, there are no risks… you can only have benefits doing exercise. (Riccardo, prostate cancer, metastases at the spine, ribs, and pelvis)

### 3.6 Theme 4: A Structured and Tailored Exercise Program Is the Key

This theme encapsulates the crucial factor for achieving benefits, minimizing potential risk, and facilitating adherence among patients living with bone metastases, that is, the features related to the exercise program. Two core sub‐themes emerged from the interviews: (i) skilled exercise specialists and (ii) tailored programs for bone metastatic patients.

#### 3.6.1 Subtheme 4.1: Skilled Exercise Specialists

Patients strongly emphasized the necessity of supervision by skilled professionals with expertise in managing individuals with bone metastases. They reported that such supervision was fundamental in creating a sense of safety, minimizing the risk of skeletal‐related adverse events, and reassuring them.

It is better to be supervised by someone with experience in this field so I can avoid mistakes that could hurt me. (Riccardo, prostate cancer, metastases at the spine, ribs, and pelvis)

From interviews, the patients' trust in exercise specialists emerged as a pivotal issue in their rehabilitation journey. Patients appreciated the ability of exercise professionals to tailor exercises to their medical history and individual physical condition and dynamically adapt their sessions, ensuring that exercises were neither too strenuous nor too risky. This adaptive approach was particularly valued for addressing variations in their daily physical condition.

The guidance we receive in performing any exercise is a sort of protection. If there is something wrong that makes me too tired, the exercise is adapted in agreement with the exercise professional. (Alberto, prostate cancer, metastases at the spine and humerus)

#### 3.6.2 Subtheme 4.2: Tailored Programs for Patients With Bone Metastases

Participants highlighted the importance of a program specifically designed for individuals with bone metastases, as it was perceived to be safer and more effective than a generalized exercise prescription. A dedicated exercise program designed for patients with cancer was seen as an environment that fostered confidence and reduced anxiety about injury or exacerbating their condition. Patients expressed a preference for exercising in a program dedicated to patients with cancer since they perceived conventional gyms as unsuitable or even hazardous. These facilities were often described as lacking both the appropriate equipment and staff knowledgeable about the unique needs of patients with cancer.

If you go to the first gym randomly, it is definitely not good… because maybe you do the wrong exercises, the program is not tailored, which makes your situation worse. (Valentina, lung cancer, metastases at the spine, pelvis, and ribs)

### 3.7 Theme 5: Personal/Interpersonal Modulators

Participants described several factors that influence their compliance and adherence to exercise programs. Key elements were grouped into four sub‐themes: (i) oncologist's recommendation, (ii) family and peer support, (iii) personal motivation, and (iv) disease and treatment side effects.

#### 3.7.1 Subtheme 5.1: Oncologist's Recommendation

The role of oncologists emerged as pivotal in encouraging patients to participate and adhere to the program. Many participants reported that their oncologist's approval served as both reassurance and motivation, as they perceived them to have the most comprehensive understanding of their physical and medical condition. This professional “validation” was often seen as a crucial prerequisite for exercise. For patients, this recommendation acted as the gateway to participation; without it, they reported they would not have considered exercising.

The medical advice surely helps to understand whether to exercise or not… since we are in a fragile condition. (Luca, colorectal cancer, metastases at the spine)

#### 3.7.2 Subtheme 5.2: Family and Peer Support

From the interviews, it appeared that family members played a dual role, both encouraging participation and sometimes unintentionally discouraging it. Participants stated that negative perceptions or lack of understanding within family members or their social circles about exercise could be barriers. For instance, some participants noted that warnings against exercise from friends or relatives undermined their confidence in exercising. On the other hand, support, such as being accompanied to sessions or exercising with family members, transformed the experience into a shared activity.

My husband used to encourage me to get outside…when I was undergoing chemo, he would go for a run, and I had to follow him on the bike every week…I remember crying because I had no energy, yet I didn't skip a single session. (Anna, breast cancer, metastases at the spine).

Exercising with other patients with cancer was reported as a facilitator for adherence to the program. The sense of camaraderie and shared understanding created a supportive environment that helped participants feel less isolated in their journey.

Exercising with others is beneficial because if you have something to share about your state of mind, you know there is someone who understands. (Alberto, prostate cancer, metastases at the spine and humerus)

#### 3.7.3 Subtheme 5.3: Personal Motivation

Personal motivation was identified as a cornerstone for starting and maintaining exercise. For many participants, the desire to regain physical function, manage symptoms, and contribute to their recovery was a strong driver. Nevertheless, participants recognized that a cancer diagnosis could profoundly impact patients' mental health, leading, in some cases, to demotivation from exercising.

One hindering factor is a kind of personal resistance…a person can become so psychologically demoralized that they no longer feel like doing anything. (Paola, lung cancer, metastases at the spine and the femur)

Prior exercise experiences emerged as a significant facilitator of engagement. Participants with a history of exercise felt more confident in their ability to adapt to new routines, while those without such experience faced greater apprehension. One participant noted that patients who have never engaged in physical activity before might not feel confident to start when a bone metastatic disease is diagnosed.

#### 3.7.4 Subtheme 5.4: Disease and Treatment Side Effects

Cancer symptoms and treatment side effects posed considerable barriers to exercise participation. Pain, particularly from bone metastases, was a recurrent theme.

Bone metastases are very painful…additionally, the treatments (hormone therapy) I take also limit my movements. (Chiara, breast cancer, metastases at the spine and pelvis)

Adverse effects due to systemic treatments, including fatigue, reduced mobility, and loss of independence, were reported as significant limitations, often resulting in an inability to drive and thus reach the exercise facilities.

### 3.8 Theme 6: Environmental/External Modulators

Environmental factors also emerged as determinants of patients' engagement in exercise programs. These factors were grouped into two subthemes: (i) facilities features and (ii) more detailed information.

#### 3.8.1 Subtheme 6.1: Facilities Features

The exercise setting emerged to have a role in encouraging participation, and the preferences vary among patients. Some participants would have preferred a hospital‐based program as medical oversight and proximity to their healthcare providers provided reassurance regarding the safety of exercise. Other participants appreciated the non‐clinical environment, viewing it as more mentally supportive and less associated with their disease.

In my opinion, exercising in the hospital is not ideal because it makes us feel like we are still undergoing therapy…exercise should be something we do for ourselves, outside. (Anna, breast cancer, metastases at the spine)

Distance to exercise facilities was also a common concern. Patients living far from dedicated centers often found travel burdensome, particularly given the fatigue and physical limitations associated with their condition.

#### 3.8.2 Subtheme 6.2: More Detailed Information

Patients stated that for implementing exercise and thus engaging more patients in the future, accessible and detailed information about the benefits of exercise for patients with bone metastases is needed. Many participants reported that, compared to other supportive care, they struggled to find reliable guidance regarding exercise and bone metastases before they participated in the program. To overcome this barrier, some participants suggested the inclusion of exercise specialists within hospital teams or providing dedicated resources to answer patients' questions and refer them to appropriate programs.

## 4 Discussion

The primary aim of our study was to gain a deeper understanding of how patients living with bone metastases perceive physical exercise. Unfortunately, these patients are often excluded from exercise programs due to safety reasons and tend to become increasingly sedentary after diagnosis [12]. To our knowledge, this is the first qualitative research exploring the experiences of patients who have attended a highly individualized exercise program aligned with the guidelines for managing bone metastases [10].

Participants described how bone metastases profoundly impacted their physical, mental, and social dimensions, including limitations in daily activities, debilitating pain, psychological impairments, and a pervasive fear of movement or skeletal‐related adverse events. This aligns with existing evidence, as bone metastases are a well‐recognized condition that can exacerbate symptom burden and impair patients' quality of life across physical, functional, and psycho‐social domains [28].

In our qualitative study, patients reported several physical benefits linked to their participation in the program, which consequently improved their ability to perform daily activities. These improvements were particularly significant as they allowed patients to resume movements that had previously been difficult due to their condition. The “*return to normality*” theme is well‐established in cancer literature following participation in exercise programs and is a strong motivation for patients to start an exercise intervention [29, 30, 31, 32]. During the interviews, patients highlighted two notable effects of the exercise intervention: the role of increased muscle mass in supporting bone structure and the potential direct benefits of exercise on bone health. Several studies confirmed that exercise can generate sufficient load to stimulate osteogenesis, and thus improve bone density and reduce fracture risk [4, 7, 33, 34]. Nevertheless, exercise is also a potent modulator of muscle mass [35, 36, 37]. Since some data reports that sarcopenia is associated with an increased mortality risk in patients with bone metastases [38], the importance of maintaining an adequate muscle mass is relevant. However, to our knowledge, no prior qualitative investigations have found these aspects making the present study particularly significant. In this sense, considering that bones are often compromised in cancer, including not only the disease‐related bone metastases but also the treatment‐related conditions, such as osteopenia and/or osteoporosis, it is essential to educate patients on the potential benefits of exercise in this context.

In our study, the most cited symptom was pain. Notably, patients recognized the benefits of exercise in reducing pain, but they also identified it as a potential barrier to starting a dedicated program. This double side of the relation between exercise and pain should not be surprising. Patients experiencing pain could be discouraged from exercising, especially for the fear of worsening their condition. Nevertheless, a randomized controlled trial involving 60 patients with spine bone metastases has demonstrated the analgesic effects of strength training, showing a reduction in pain levels and a decreased use of opioid and non‐opioid medications after 12 weeks of exercise [7]. Pain is the most common symptom reported by 64% of patients with bone metastases, and thus, finding suitable ways to promote exercise in this context is fundamental [2]. In this sense, informing patients about the positive impact of physical exercise on this outcome could be a first step delivered by clinicians to encourage patients to incorporate exercise into their care plans. At the same time, it is crucial, as it has been found in our study, that the program should be supervised by dedicated experts equipped to monitor pain and adjust exercise prescriptions as needed, taking into account psychological factors such as fear of movement and injuries.

Patients expressed safety concerns by exercising alone, without the supervision of exercise professionals, fears reported in prior studies exploring the experiences of patients with advanced or metastatic cancer regarding exercise [9, 29, 31]. In this sense, the involvement of exercise specialists, physiotherapists, or nurses is often associated with a sense of safety, and it is recognized as a key facilitator for exercise engagement [9, 14]. This is also highlighted by our participants, who reported that a crucial positive factor was the supervision of the program by dedicated experts trained to tailor exercise to the medical history and adjust prescriptions as needed, taking into account psychological factors such as fear of movement and risk of injuries. However, some participants preferred more independent exercise in less supervised environments, highlighting the need to offer diverse exercise options that accommodate both preferences. Indeed, several studies report the feasibility of the exercise in the context of bone metastases, especially supervised, and highlight the absence of serious skeletal‐related adverse events [4, 5, 15].

Among the modulators for exercise engagement identified by the patients, beyond personal motivation, previous exercise history, and disease‐related issues that have been extensively reported from the literature, there was a strong consensus among participants regarding the need for the oncologist's advice for exercising [39]. This has been perceived as a pivotal factor, strongly favoring motivation toward exercise. This could be explained by the fact that patients viewed their oncologists as the most credible source of advice, with many stating they would not have started exercising without a direct referral. This aligns with results found in a cross‐sectional study on 549 patients with cancer, which revealed that 74% of them think that exercise is an issue that should be discussed with the oncologists [40]. Nevertheless, the study further noted that oncologists were more likely to refer patients with early‐stage disease to exercise programs, likely due to their lower symptom burden. This trend suggests that despite evidence supporting the benefits and safety of exercise, a gap in exercise promotion for patients with advanced or metastatic disease and referrals remains limited for this population. Addressing this gap requires a systemic approach to integrating exercise education and resources into oncology care. Including exercise specialists within the hospital team could bridge this divide by providing personalized advice, dispelling misconceptions about exercise safety, and offering structured guidance tailored to individual patient needs. Such efforts could increase patient participation in exercise programs and ensure they derive the well‐documented physical and psychological benefits of physical exercise.

Participants noted that encouragement from family members helped them initiate and sustain an exercise routine. Conversely, a lack of support or skepticism from family members posed a significant barrier. These findings underscore the importance of involving caregivers in educational initiatives to promote a shared understanding of the benefits and safety of exercise for patients with bone metastases. Social support, especially from peers, was also a valuable facilitator for participants, rendering the exercise sessions more enjoyable and providing a way to share experiences. The social component of exercise interventions is frequently reported as a positive aspect by patients participating in structured exercise programs as it fosters a sense of connection and contributes to a sense of normalcy. These findings are consistent with the study of Bland et al., who explored the perception of exercise in patients with advanced cancer and cachexia. Patients viewed spouses and other patients as sources of encouragement and considered the group‐based program as a valuable option [31]. Environmental factors, such as the facility's features, significantly influenced exercise participation, and patients showed different preferences. Some participants preferred non‐clinical settings, associating them with greater autonomy and a sense of normalcy. Others valued hospital‐based settings for the perceived safety and supervision they provided. These contrasting preferences underscore the need for diverse program options, accommodating both those who seek medical oversight and those who prefer non‐clinical environments. Additionally, logistical barriers such as distance to facilities were frequently cited, emphasizing the importance of offering accessible and flexible solutions, including telehealth‐based or home‐based interventions.

### 4.1 Strengths and Limitations

Our study contributes significantly to cancer and exercise literature for several reasons. To our knowledge, this study is the first that has explored the perception of patients with bone metastatic cancer following their participation in a supervised exercise program. Prior investigations involved exclusively patients with metastatic breast cancer, in which only a subset was affected by bone lesions, and participants were mixed in terms of exercise program engagement [9, 14, 16], making our investigation unique in these terms. Regarding the methodology, we used inductive thematic analysis to gather rich information from patients with mixed characteristics. The rigorous data collection and interpretation facilitate the potential transferability of our conceptual model to a broader context.

Despite these strengths, our study has some limitations. A potential limitation of this study relates to researcher positionality and its possible influence on data interpretation. While measures were taken to minimize bias, complete neutrality cannot be guaranteed, particularly given the clinical and supportive roles some researchers held in patient care. Whereas the heterogeneity in terms of sex, age, cancer types, and treatments enhances generalizability, the relatively high level of education among participants may introduce a selection bias. Furthermore, as our study exclusively included individuals who had completed a structured exercise program, the findings likely reflect the perspectives of more motivated and exercise‐oriented individuals. This focus may limit the applicability of the results to less motivated populations or those naive to structured exercise programs. Future research should explore the perspectives of patients with bone metastases who have not participated in exercise interventions, as they may perceive exercise as unsafe or unnecessary and may lack awareness of its potential benefits. Understanding both perspectives will be crucial in identifying barriers, needs, and misconceptions, ultimately helping to develop strategies that encourage broader participation in exercise among patients with bone metastases.

### 4.2 Clinical Implications

This study highlights several important clinical considerations. Patients perceived several benefits by participating in a structured physical exercise program, encompassing physical fitness, symptoms, and psychological well‐being. Whereas patients highlighted the structured physical exercise program designed for individuals affected by bone metastases as the key to achieving benefits, they also individuated a series of modulators that may influence their participation. These findings emphasize the need for oncology care teams, particularly oncologists, to proactively recommend exercise to patients with bone metastases, reassuring them of its safety when appropriately tailored and supervised. Integrating exercise counseling into routine oncology care, possibly through multidisciplinary collaborations with exercise specialists and psychologists, could enhance patient engagement and adherence. Additionally, educating families about the role of exercise may provide patients with both practical and emotional support, further facilitating participation.

## 5 Conclusion

Participants provided unique perspectives about their participation in a structured exercise program. Bone metastases significantly impacted patients' lives, causing limitations, pain, and fear of movement. These challenges were alleviated through tailored exercise programs, helping patients regain a sense of control. Whereas a supervised and tailored exercise program is fundamental, several personal and external modulators may influence exercise adherence.

## Author Contributions

**Anita Borsati:** conceptualization (equal), data curation (equal), formal analysis (equal), investigation (equal), methodology (equal), visualization (equal), writing – original draft (equal), writing – review and editing (equal). **Daniela Tregnago:** formal analysis (equal), investigation (equal), methodology (equal), resources (equal), validation (equal), visualization (equal), writing – review and editing (equal). **Matteo Caleffi:** data curation (equal), formal analysis (equal), investigation (equal), methodology (equal), resources (equal), writing – review and editing (equal). **Christian Ciurnelli:** investigation (equal), visualization (equal), writing – review and editing (equal). **Linda Toniolo:** investigation (equal), visualization (equal), writing – review and editing (equal). **Ilaria Trestini:** resources (equal), visualization (equal), writing – review and editing (equal). **Lorenzo Belluomini:** resources (equal), visualization (equal), writing – review and editing (equal). **Marco Sposito:** resources (equal), visualization (equal), writing – review and editing (equal). **Jessica Insolda:** resources (equal), visualization (equal), writing – review and editing (equal). **Federico Schena:** resources (equal), supervision (equal), visualization (equal), writing – review and editing (equal). **Michele Milella:** conceptualization (equal), data curation (equal), project administration (equal), supervision (equal), validation (equal), visualization (equal), writing – review and editing (equal). **Sara Pilotto:** conceptualization (equal), data curation (equal), project administration (equal), supervision (equal), visualization (equal), writing – original draft (equal), writing – review and editing (equal). **Alice Avancini:** conceptualization (equal), data curation (equal), formal analysis (equal), investigation (equal), methodology (equal), project administration (equal), resources (equal), software (equal), supervision (equal), validation (equal), visualization (equal), writing – original draft (equal), writing – review and editing (equal).

## Ethics Statement

This study was performed in line with the principles of the Declaration of Helsinki and Oviedo. The project was reviewed and approved by the Ethics Committee for Clinical Trials of the University of Verona (Prot. N. 33320).

## Consent

Written informed consent and consent to publish were obtained from all study participants.

## Conflicts of Interest

S.P. reports personal fees from AstraZeneca, Eli‐Lilly, Novartis, AMGEN, BMS, Boehringer Ingelheim, Merck & Co., and Roche and grants from AstraZeneca and BMS outside of the submitted work. M.M. reports personal fees from Pfizer, MSD, AstraZeneca, EUSA Pharma, Boehringer Ingelheim, and Ipsen and grants from Roche and BMS outside of the submitted work. All remaining authors have no conflicts of interest to disclose.

## Supporting information


Data S1.

## Data Availability

The data that support the findings of this study are available on request from the corresponding author. The data are not publicly available due to privacy or ethical restrictions.
