# Supplementary material for: Overcome the Fear of Exercise in Patients With Bone Metastases: A Qualitative Study on Patients' Perception
Source: Cancer Med. 2025 Apr 17;14(8):e70865. doi: 10.1002/cam4.70865 (PMC12004276; doi:10.1002/cam4.70865)
Supplement: Supplementary file 1 — Data S1. [file CAM4-14-e70865-s001.docx]

**Supplementary Material**

**Supplementary file 1.** Inclusion and exclusion criteria for participation

**Table S1.** Exercise program characteristics

**Table S2.** Themes, subthemes and illustrative quotes emerged from the interviews

**Supplementary file 1.** Inclusion and exclusion criteria for participation

**Inclusion criteria:**

- Age ≥ 18 years
- Histologically confirmed diagnosis of stage IV solid cancer
- Presence of one or more osteolytic or osteoblastic bone metastases
- Eastern Cooperative Oncology Group (ECOG) performance status ≤ 2
- Medical clearance to participate in an exercise program
- Provision of written informed consent to participate

**Exclusion criteria:**

- Major surgery within 2 months prior to enrollment
- Scheduled surgery within the 3-month exercise intervention period

**Table S1.** Exercise program characteristics

|  | Warm-up | Aerobic training | Resistance training | Cool-down |
| --- | --- | --- | --- | --- |
| Frequency | 2/week | 2/week | 2/week | 2/week |
| Intensity | Light (1-2 rate of perceived exertion C-10) | Moderate (3-5 rate of perceived exertion C-10) | Moderate (3-5 rate of perceived exertion C-10) | Light (1-2 rate of perceived exertion C-10) |
| Type | Dynamic stretching for joint mobility | Treadmill or bike | 6 body weight or elastic bands exercises individually adjusted (e.g. squat, row, push press, calf and abdomen muscles, balance exercise) | Static stretching of major muscle groups |
| Time | 5 minutes | 10-15 up to 25-30 minutes | 2-3 sets of 8-12 reps | Hold each position for 30 seconds |
| Progression | No | Yes, every two weeks | Yes, every two weeks | No |
| Precautions | Avoiding movements (e.g. rotations, twists, rapid flexions or extensions) involving bone lesions sites | Preferring activities that stimulate the osteogenesis (e.g. walking), while opting for cycling when there is a high risk of falling | - Starting with moderate or no loads exercises and progress slowly - Avoiding rapid or heavy movements that stress bone lesions - Stopping or modify exercise in presence of pain particularly at the site of metastases - Adjusting exercise technique and posture alignment - Monitoring the patient's response to exercise | Avoiding positions that exert compressive forces on the bone lesions |

**Table S2**. Themes, subthemes and illustrative quotes emerged from the interviews

| Themes and subthemes | Illustrative quotes |
| --- | --- |
| Theme 1: Bone metastatic disease affects patient’s lives | |
| Limitations in daily living and fear of injuries | *You’re afraid to make a wrong movement, so you stay still, which is worse. I did this too. I stayed still because I thought, 'If I do this, maybe I'll get worse,' but instead, you weaken even more* (Valentina, lung cancer, metastases at the spine, pelvis, and ribs)  *At the beginning, you completely rule out physical activity because the pain is so intense that any movement feels impossible* (Angelica, lung cancer, metastases at the spine, pelvis and femur) |
| Theme 2: A physical exercise program may reinforce the body and mind | |
| Improvement in physical fitness, symptoms and activities of daily living | *For me, exercise is essential… I had difficulty in breathing, pain in my leg muscles, tremors... whereas when I was regular (with exercise), I did not have these problems* (Paola, lung cancer, metastases at the spine and femur)  *It becomes easier to tie your shoes, get in and out of the car, and walk* (Alberto, prostate cancer, metastases at the spine and humerus)  *Even though you have the bone lesion, it helps improve your posture and allows you to do things you couldn't do before* (Anna, breast cancer, metastases at the spine) |
| Not letting bone metastases stop you: the psycho-social well-being | *It also helps on a psychological level; you are motivated to perform something that also offers psychological support... I feel my fatigue decreasing, and, therefore, I am more encouraged to continue* (Francesca, lung cancer, metastases at the spine)  *It helps to feel not sick... before cancer, I was usually a super-active person. After my disease, I sat all day… this can lead to depression* (Sara, lung cancer, metastases at the spine)  *Training with others and sharing experiences with people who have the same condition is helpful* (Maria, breast cancer, metastases at the spine and femur) |
| Theme 3: Exercising may impact bone health | |
| Amelioration of pain caused by bone metastases | *Thanks to exercise, the pain became much more tolerable* (Chiara, breast cancer, metastases at the spine and pelvis)  *Regarding pain… it provides significant relief, especially for those who, like me, struggle with back pain* (Anna, breast cancer, metastases at the spine) |
| Enhancement of bone health | *I think movement is also good for improving muscle mass, and as a result, it helps reduce the strain on my bones since they are fragile* (Riccardo, prostate cancer, metastases at the spine, ribs and pelvis)  *I'm not sure if there is a direct benefit for my bones because many movements have been restricted. However, aside from the spine, which is better to avoid, I believe it's beneficial for my arms and legs* (Paola, lung cancer, metastases at the spine and the femur) |
| Risk of increased skeletal-related adverse events and disease progression | *It could be that in some cases, exercise is even contraindicated because it might worsen the metastases or cause them to spread* (Maria, breast cancer, metastases at the spine and femur)  *Considering the exercise specialists who guide me, I don’t see any risks… the only risk would be if I used the resistance machines incorrectly* (Alberto, prostate cancer, metastases at the spine and humerus) |
| Theme 4: A structured and tailored exercise program is the key | |
| Skilled exercise specialists | *If I did exercise at home, I know I would make some mistakes; for me, it’s important to be supervised* (Anna, breast cancer, metastases at the spine)  *Specialized exercise specialist are needed; you can’t do whatever you want* (Elisa, breast cancer, metastases at the ribs) |
| Tailored programs for patients with bone metastases | *Going to a place where someone is either untrained or trained differently is a risk... who suggests exercises that I’m not sure are good for me or not* (Valentina, lung cancer, metastases at the spine, pelvis and ribs)  *Having bone fragility excludes you from generic exercise programs and local gyms* (Angelica, lung cancer, metastases at the spine, pelvis and femur) |
| Theme 5: Personal/interpersonal modulators |  |
| Oncologist’s recommendation | *It’s important that it’s the oncologist who recommends it because they are the point of reference. If they hadn’t told me, I wouldn’t have done anything* (Francesca, lung cancer, metastases at the spine)  *The ideal situation would be for the oncologist to recommend exercise immediately after diagnosis, when all the treatments begin... either by discussing it or perhaps with brochures* (Andrea, prostate cancer, metastases at the spine, pelvis and femur) |
| Family and peer support | *It becomes a general fear among family members... everyone is afraid that you're no longer able to do things. Even my husband was worried* (Chiara, breast cancer, metastases at the spine and pelvis)  *My wife is a pillar for me... she is very attentive to making sure that everything that can be done for my psychophysical condition is done* (Riccardo, prostate cancer, metastases at the spine, ribs and pelvis) |
| Personal motivation | *Personal motivation is needed... I do it for myself, I impose it on myself to try to overcome this fatigue, to feel a bit more alive* (Elisa, breast cancer, metastases at the ribs)  *A person who has never exercised in their life... if they develop a condition like cancer, I think they won’t begin, and they might resist engaging in physical activity* (Andrea, prostate cancer, metastases at the spine, pelvis and femur) |
| Disease and treatment side effects | *The decision to start physical exercise always depends on the type of lesion and how much pain is felt* (Luca, colorectal cancer, metastases at the spine)  *At the beginning, when I was really unwell, I was lying on the couch; I couldn’t exercise, I didn’t have the physical strength* (Maria, breast cancer, metastases at the spine and femur) |
| Theme 6: Environmental/external modulators |  |
| Facilities features | *If there were facilities outside the hospital environment, that would be great; in fact, there might even be more people... it feels less like a 'sick' environment* (Sara, lung cancer, metastases at the spine)  *If the exercise program is done within a healthcare setting, it's as if you have the certainty that it's the right thing for you* (Maria, breast cancer, metastases at the spine and femur) |
| More detailed information | *It is difficult for a patient to ask about physical activity given their current situation… unlike psychological support, which is widely recognized as complementary to cancer treatment, exercise might be perceived as painful or counterproductive without medical guidance* (Luca, colorectal cancer, metastases at the spine)  *Without medical advice, a patient wouldn't consider exercising because they think it would be painful and counterproductive. Proper information is needed* (Angelica, lung cancer, metastases at the spine, pelvis and femur) |
